# The New England Journal of Dentistry

**Published:** 1882-02

**Authors:** 


*The New England Journal of Dentistry and Allied Sciences.*—Edited by Associated Dentists. Chas. Mayr, A. M., B. S., Scientific editor. Published monthly by the New England Journal Company, Springfield, Mass.

No. 1 of Volume 1 contains original articles on Vital Force, Results of Arsenical Application to Soft Teeth, Selections, Abstracts, Editorials, and Notices of Society Meetings. Although of pamphlet form, and consisting of some thirty-two pages, the first number presents a handsome appearance, and is printed on good paper in clear type. We offer our sincere wishes for the prosperity of this new addition to dental journalism.

